# Neighborhood Income Disparities in Unplanned Hospital Admission and In-Hospital Outcomes Among Children with Congenital Heart Disease

**DOI:** 10.1007/s00246-024-03755-8

**Published:** 2024-12-26

**Authors:** Laxmi V. Ghimire, Sagya Khanal, Zareh Torabyan, Hiba El-Rahi, Catherine Cong, Fu-Sheng Chou, Othman A. Aljohani, Anita J. Moon-Grady

**Affiliations:** 1 Division of Pediatric Cardiology, Department of Pediatrics, University of California, San Francisco, Fresno Regional Campus, 215 N Fresno St, Fresno, CA USA; 2 Nepal Medical College, Kathmandu, Nepal; 3 Department of Pediatrics, University of California, San Francisco, Fresno Campus, Fresno, CA USA; 4 Department of Neonatal-Perinatal Medicine, Kaiser Permanente Riverside Medical Center, Riverside, CA USA; 5 Division of Pediatric Cardiology, Benioff Children’s Hospital, Department of Pediatrics, University of California, San Francisco (UCSF), San Francisco, CA USA

**Keywords:** Congenital heart disease, Unplanned admission, Elective admission, Socioeconomic disparity, United States, ICD-10 codes

## Abstract

Unplanned admissions are associated with worse clinical outcomes and increased hospital resource utilization. We hypothesized that children with congenital heart disease (CHD) from lower-income neighborhoods have higher rates of unplanned hospital admissions and greater resource utilization. Utilizing the Kids’ Inpatient Database (2016 and 2019), we included children under 21 years of age with CHD, excluding newborn hospitalizations. CHD cases were categorized into simple lesions, complex biventricular lesions, and single ventricle lesions. Admissions were classified as surgical or non-surgical. A logistic regression model assessed the risk of unplanned hospital admission, mortality, and resource utilization across different neighborhood income levels. Out of 4,722,684 admitted children (excluding newborn hospitalizations), 199,757 had CHD and met the study criteria: 121,626 with mild CHD, 61,639 with complex biventricular lesions, and 16,462 with single ventricle lesions. Surgical admissions comprised 20% (n = 39,694). In the CHD cohort, 27% had planned admissions, while 73% were unplanned. Mortality was higher in unplanned admissions compared to planned admissions (3.0 vs. 0.93%, P < 0.001). Unplanned admissions were more common in the lowest-income neighborhoods compared to the highest-income neighborhoods (adjusted odds ratio [aOR] = 1.4; 95% confidence interval [CI]: 1.3–1.5; P < 0.001), consistent across different age groups. Higher rates of unplanned admissions in the lowest-income neighborhoods were observed for each CHD category and for both medical and surgical admissions. Median hospitalization length was longer in the poorest neighborhoods compared to the wealthiest (7 days [IQR 3–21] vs. 6 days [IQR 3–17], P < 0.001). In conclusion, children with CHD residing in the lowest-income neighborhoods have increased odds of unplanned hospitalization for both surgical and non-surgical admissions, along with higher mortality and resource utilization.

## Introduction

Congenital heart disease (CHD) is the most prevalent congenital anomaly, affecting approximately 1% of the population and significantly contributing to pediatric morbidity and mortality [1, 2]. While the incidence of CHD is relatively consistent across various geographic, racial, and socioeconomic groups, disparities in access to care and health outcomes have been documented [3–5]. Socio-economic factors play a pivotal role in determining the healthcare access and outcomes for individuals with heart disease [6, 7]. Studies have shown that individuals with limited resources and healthcare access experience worse outcomes [8–12]. Socioeconomic disparities in healthcare utilization—such as delayed diagnosis, suboptimal management, and higher complication rates—contribute to the unequal disease burden in vulnerable populations [9, 10, 13]. A recent meta-analysis of 28 studies by Best et al. indicated that lower socioeconomic status is associated with increased short-term and long-term mortality among neonates, infants, and children with CHD [14]. National and regional data have explored disparities in outcomes based on race and socioeconomic conditions in CHD populations [15–23]. Several predominantly single-center studies have reported that low socioeconomic status, Hispanic ethnicity, younger age, and higher complexity of CHD lesions are associated with increased readmissions and higher mortality after CHD surgery [24–27]. However, to our knowledge, there are no reports on national or large-scale regional data evaluating the role of socioeconomic status in CHD admission types and subsequent outcomes. Utilizing the latest datasets from the Kids’ Inpatient Database (KID), this study aims to bridge this knowledge gap and provide insights into the influence of neighborhood income level on unplanned/planned hospital admissions and clinical outcomes in children with CHD.

## Methods

We analyzed data from the Kids’ Inpatient Database (KID) for the years 2016 and 2019 [28, 29]. The KID, developed by the Agency for Healthcare Research and Quality (AHRQ) as part of the Healthcare Cost and Utilization Project (HCUP), is a cross-sectional database that includes discharge data from a representative sample of U.S. hospitals. Each iteration of the KID encompasses data from over three million annual births and six million hospital discharges, with publications every three years; 2019 is the most recent release [29]. Our study population comprised children up to 20 years of age. We excluded all newborn hospitalizations, including those where infants underwent surgery during their initial birth admission, regardless of whether the surgery occurred at the birth hospital or after transfer to a cardiac center. The KID employs a stratified, cross-sectional design, capturing approximately 10% of newborn discharges and 80% of other discharges nationwide. We applied sample weights to patient-level discharge observations to generate nationally representative estimates of U.S. hospitalizations.

Neighborhood ZIP Codes were used to classify the estimated median household income of residents into quartiles, ranging from the lowest to highest income neighborhoods. These values were derived from ZIP Code demographic data obtained from Claritas [30].

To identify children with CHD, we used the International Classification of Diseases, Tenth Revision, Clinical Modification (ICD-10-CM) codes. We classified the CHD cases into three categories based on complexity: simple lesions, complex biventricular lesions, and single ventricle lesions. Simple/mild CHD included atrial septal defect (ASD), ventricular septal defect (VSD), patent ductus arteriosus (PDA) and other septal defects. Single ventricle lesions included hypoplastic left heart syndrome, tricuspid atresia/stenosis, aortic atresia/stenosis and common ventricle. The remaining lesions were classified as biventricular complex lesions as per the previous classification, which has been previously used in the pediatric cardiology literature [31]. We identified children who underwent surgical repair during hospitalization using ICD-10 procedure codes for CHD surgical interventions. Age groups were categorized as < 1, 1–5, 6–10, and 11–20 years. In the KID database, admission types are classified as non-elective(unplanned) and elective(planned).

The primary objective was to assess the rate or risk of unplanned hospital admissions in children with CHD from the lowest-income neighborhoods compared to those from the highest-income neighborhoods. Secondary outcomes included mortality rates in CHD admissions across different income quartiles, stratified by unplanned and planned admissions, as well as surgical and non-surgical categories. We also examined the length of hospital stay as a secondary outcome.

Descriptive statistics were performed to summarize the characteristics of the study population. Categorical variables were presented using frequencies and percentages, while continuous variables were expressed as median and interquartile range (IQR). To compare categorical variables, we employed the chi-square test.

To evaluate the association between neighborhood income level and the odds of unplanned admission, we conducted logistic regression analysis. Neighborhoods were classified into four groups based on income levels, from lowest to highest income. Adjustments were made for confounding variables, including age, sex, year of admission, presence of extracardiac anomalies(ECA) and CHD complexity. Race/ethnicity was excluded from the analysis due to approximately 10% missing data, and HCUP advises against its use in regular analysis.

Weights provided by HCUP were used in all analyses to account for the complex sampling design and clustering for the analysis. All statistical analyses were conducted using Stata version 14 (StataCorp, College Station, TX, USA) and R-rstudio (R Foundation for Statistical Computing, Vienna, Austria). The complex survey design of the KID was accounted for using the *survey* package [32].

This study utilized a publicly available de-identified database and received expedited institutional review board approval from the Community Regional Medical Center, University of California San Francisco, Fresno campus, CA, USA.

## Results

The study analyzed 4,722,684 pediatric admissions, excluding newborn hospitalizations, identifying 199,757 cases of congenital heart disease (CHD). Among these, 121,626 were classified as mild CHD, 61,639 as complex biventricular lesions, and 16,492 as single ventricle lesions. Surgical admissions accounted for 20% (n = 39,694) of the CHD cases, with the remainder being non-surgical. 39% (n = 47,306) of children with CHD have extracardiac anomalies(ECA).

Unplanned admissions constituted 79% of all non-newborn pediatric hospitalizations and 73% within the CHD cohort. Within the CHD cohort, unplanned hospitalizations were more prevalent in the lowest-income neighborhoods (76%) compared to the highest-income neighborhoods (68%) (p < 0.001) Table 1. Logistic regression analysis, adjusted for covariates, indicated that children with CHD from the lowest-income neighborhoods had higher odds of unplanned hospitalization than those from the highest-income neighborhoods (adjusted odds ratio [aOR] = 1.4; 95% confidence interval [CI]: 1.3–1.5; p < 0.001) Table 3. This trend persisted across both surgical and non-surgical groups, with higher unplanned admissions in the lowest-income neighborhoods compared to the highest-income neighborhoods Table 2.

**Table 1:** Unplanned and elective admission types in total non-newborn pediatric hospitalization and those with CHD cohort

Household zipcodes	All children* (N = 4,722,684)		CHD only children^#^(N = 199,757)	
	Unplanned/non-elective	Elective	Unplanned/non-elective	Elective
0–24th	1,247,954(79%)	327,178(21%)	48,564(76%)	15,295(24%)
25–49th	924,618(78%)	256,205(22%)	36,951(74%)	13,288(26%)
50–74th	868,009(79%)	227,835(21%)	34,443(72%)	13,542 (28%)
75–100th	694,240(80%)	176,647(20%)	25,724(68%)	11,950(32%)
Total	3,734,819(79%)	987,865(21%)	145,683(73%)	54,074(27%)

*P = 0.0116, ^#^P < 0.001

**Table 2:** Unplanned and elective admission types in total non-newborn CHD cohort, according to surgical and non-surgical admission

Household zipcodes	Surgical admission*(n = 39,694)		Non-surgical admission^#^(n = 160,063)	
	Unplanned/non-elective	Elective	Unplanned/non- elective	Elective
0–24th	4157(37%)	7179(63%)	44,407(85%)	8116(15%)
25–49th	3358(34%)	6405(66%)	33,594(83%)	6883(17%)
50–74th	3206(32%)	6684(68%)	31,237(82%)	6858 (18%)
75–100th	2681(31%)	16,024(69%)	23,043(80%)	5925(20%)
Total	13,402(34%)	26,292(66%)	132,281(83%)	27,782(17%)

*P < 0.001, ^#^P < 0.001

Unplanned hospitalization rates varied by CHD category: 79% in simple CHD, 64% in complex biventricular lesions, and 63% in single ventricle lesions, P < 0.001. Across all CHD categories, the risk of unplanned hospitalization was consistently higher in the lowest-income neighborhoods compared to the highest-income neighborhoods Table 3.

**Table 3:** Logistic regression of unplanned admissions in different zip codes (unadjusted and adjusted) in CHD subpopulation (N = 199,656). Adjusted for age, sex, year and extracardiac anomalies

Household zipcodes	Emergent/Non-Elective	Unadjusted OR	P-value	Adjusted OR	P-value
All CHD					
0–24th	48,564(76%)	1.5(1.4–1.6)	< 0.001	1.4(1.3–1.5)	< 0.001
25–49th	36,951(74%)	1.3(1.2–1.4)	< 0.001	1.2(1.2–1.3)	< 0.001
50–74th	34,443(72%)	1.2(1.1–1.2)	< 0.001	1.1(1.1–1.2)	< 0.001
75–100th	25,724(68%)	Reference	-	Reference	-
CHD category 1					
0–24th	33,100(82%)	1.5(1.4–1.7)	< 0.001	1.4(1.3–1.6)	< 0.001
25–49th	24,363(79%)	1.3(1.2–1.4)	< 0.001	1.2(1.1–1.3)	< 0.001
50–74th	22,361(78%)	1.2(1.1–1.3)	< 0.001	1.2(1.1–1.3)	< 0.001
75–100th	16,191(75%)	Reference	-	Reference	-
CHD category 2					
0–24th	12,279(67%)	1.4(1.3–1.5)	< 0.001	1.4(1.3–1.5)	< 0.001
25–49th	9891(65%)	1.3(1.2–1.4)	< 0.001	1.3(1.2–1.4)	< 0.001
50–74th	9597(63%)	1.2(1.1–1.2)	< 0.001	1.1(1.1–1.2)	< 0.001
75–100th	7566(59%)	Reference	-	Reference	-
CHD category 3					
0–24th	3186(64%)	1.1(1.1–1.3)	0.037	1.1(1.0–1.3)	0.051
25–49th	2697(63%)	1.1(1.0–1.2)	0.24	1.1(0.9–1.2)	0.26
50–74th	2485(61%)	1.0(0.9–1.1)	0.72	1.0(0.9–1.1)	0.69
75–100th	1968(62%)	Reference	-	Reference	-

*CHD* Congenital heart disease

Analysis across different age groups (0–20 years) revealed that the risk of unplanned admissions was higher in each age group for children from the lowest-income neighborhoods compared to those from the highest-income neighborhoods Table 4.

**Table 4:** Logistic regression of CHD non-elective/emergent admissions in different zip codes (according to age group) Adjusted for sex, year and extracardiac anomalies

Household zipcodes	Emergent/Non-Elective	Unadjusted OR	P-value	Adjusted OR	P-value
Age group: < 1 year					
0–24th	33,927(83%)	1.4(1.2–1.5)	< 0.001	1.4(1.2–1.5)	< 0.001
25–49th	25,454(81%)	1.2(1.1–1.3)	< 0.001	1.2(1.1–1.3)	< 0.001
50–74th	22,883(80%)	1.1(1.1–1.2)	< 0.001	1.1(1.05–1.2)	< 0.001
75–100th	16,813(78%)	Reference	-	Reference	-
Age group: 1–5 years					
0–24th	8651(65%)	1.4(1.3–1.6)	< 0.001	1.4(1.3–1.6)	< 0.001
25–9th	6743(63%)	1.3(1.2–1.4)	< 0.001	1.3(1.2–1.4)	< 0.001
50–74th	6578(61%)	1.2(1.1–1.3)	< 0.001	1.2(1.1–1.3)	< 0.001
75–100th	4665(56%)	Reference	-	Reference	-
Age group: 6–10 years					
0–24th	2251(58%)	1.4(1.2–1.6)	< 0.001	1.4(1.2–1.7)	< 0.001
25–49th	1650(54%)	1.2(1.0–1.3)	0.007	1.2(1.1–1.4)	0.004
50–74th	1849(56%)	1.3(1.1–1.4)	< 0.001	1.3(1.1–1.4)	< 0.001
75–100th	1384(50%)	Reference	-	Reference	-
Age group: 11–20 years					
0–24th	3735(63%)	1.4(1.2–1.5)	< 0.001	1.4(1.2–1.6)	< 0.001
25–49th	3104(61%)	1.2(1.1–1.4)	0.001	1.2(1.1–1.4)	0.001
50–74th	3133(59%)	1.1(1.0–1.3)	0.026	1.1(1.0–1.3)	0.03
75–100th	2862(55%)	Reference	-	Reference	-

*CHD* Congenital heart disease

Extracardiac anomalies (ECAs) were more prevalent among children from lower-income families, with rates of 40% in the lowest-income neighborhoods compared to 37% in the highest-income neighborhoods (p < 0.001). A multivariable logistic regression model indicated that the presence of ECAs in complex CHD categories (Category 2 and Category 3) is associated with a higher risk of unplanned hospital admissions.

Children with CHD from the lowest-income neighborhoods exhibited higher mortality rates (2.6%, n = 1,673) compared to those from the highest-income neighborhoods (2.1%, n = 798), p = 0.0012 Table 5. Mortality rates were higher in the surgical group compared to the non-surgical group within the unplanned admissions. Although higher mortality rates were observed in lower-income neighborhoods for both surgical and non-surgical unplanned admissions, these differences were not statistically significant Table 6. Unplanned admissions had a threefold higher mortality rate compared to planned admissions within the CHD cohort (3.0%, n = 4,380 vs. 0.93%, n = 513; p < 0.001). In planned admissions, children from the lowest-income neighborhoods had nearly double the mortality rate compared to those from the highest-income neighborhoods 1.2% (n = 181) vs. 0.65%(n = 78); aOR = 1.7 (95% CI 1.2–2.4), p = 0.005). Table 7. In the unplanned admission group, the mortality rate was 11% higher in the lowest-income neighborhoods compared to the highest-income neighborhoods (3.1 vs. 2.8%), though this difference was not statistically significant.

**Table 5:** Mortality according to zip code for all non-newborn pediatric and CHD admissions

In-hospital mortality	Household income zip code quartile				
	0–24th	25–49th	50–74th	75–100th	
All pediatric hospitalization(Population size = 4,719,922)					
No	1,566,795	1,174,353	1,089,907	866,338	P = 0.0018
Yes	7785(0.49%)	5698(0.48%)	5237(0.48%)	3809(0.44%)	
CHD only cohort (Population size = 199,656)					
No	62,164	48,988	46,847	36,847	
Yes	1673(2.6%)	1230(2.4%)	1109(2.3%)	798(2.1%)	P = 0.0012

**Table 6:** Mortality according to ZIP code for all non-newborn CHD non-elective admissions(surgical and non-surgical)

In-hospital mortality	Household income zip code quartile				
	0–24th	25–49th	50–4th	75–100th	
Surgical CHD non-elective admission (Population size = 13,391)					
No	3953(95%)	3197(95%)	3061(95%)	2551(95%)	P = 0.93
Yes	201(4.8%)	161(4.8%)	145(4.5%)	122(4.6%)	
Nonsurgical CHD non-elective admission (Population size = 132,204)					
No	43,099	32,633	30,357	22,430	
Yes	1291(2.9%)	944(2.8%)	851(2.7%)	598(2.6%)	P = 0.28

**Table 7:** Mortality by ZIP code in CHD children in unplanned and elective admissions

In-hospital mortality	Household income zip code quartile				
	0–24th	25–49th	50–74th	75–100th	P-value
Non-elective admission					
No	47,051(97%)	35,830(97%)	33,419(97%)	24,981(97%)	0.37
Yes	1493(3.1%)	1105(3.0%)	996(2.9%)	720(2.8%)	
Elective admission					
No	15,113(99%)	13,158(99%)	13,428(99%)	11,866(99%)	P = 0.0061
Yes	181(1.2%)	125(0.94%)	112(0.83%)	78(0.65%)	

Children with CHD from the lowest-income neighborhoods experienced longer median length of stay (LOS) during unplanned hospital admissions (7 days; interquartile range [IQR]: 3–21) compared to those from the highest-income neighborhoods (6 days; IQR: 3–17), p < 0.001.

## Discussion

This study aimed to explore the association between income status, as measured by neighborhood ZIP codes, and types of hospital admissions in children with congenital heart disease (CHD), along with their outcomes. The findings reveal significant disparities in hospital admission rates, mortality rates, and lengths of stay associated with the income status of patients’ neighborhoods.

A major finding was that children with CHD from the lowest-income neighborhoods experienced higher rates of unplanned hospital admissions across all CHD categories and age groups. This suggests that family income plays a crucial role in access to timely and appropriate healthcare. Tregay et al. [24] conducted a meta-analysis of 15 single-center studies, revealing that lower SES correlates with a higher risk of unplanned admissions, a finding consistent with our results. Their study also identified non-white race and complex CHD as factors associated with increased readmission risk. However, due to limitations in the KID database—specifically, over 10% missing data on race—we were unable to analyze the impact of race on outcomes in our study.

Benavidez et al. examined 8585 index admissions and found that racial minority status, complex CHD, and government insurance (used as a proxy for lower SES) were linked to higher rates of unplanned admissions [25]. Similarly, Lushaj et al. studied 120 readmission cases and reported that lower family income, younger age, complex heart disease, and prolonged previous hospitalizations were associated with an elevated risk of unplanned admissions [26].

Our study extends these findings to a national level by utilizing the KID database. This broader scope allows for a more comprehensive understanding of the relationship between SES, admission types, and outcomes in the CHD population.

Best et al.’s meta-analysis, encompassing five studies on socioeconomic disparities in congenital heart disease (CHD) outcomes, identified a clear association between lower socioeconomic status and poorer outcomes across various CHD severities and age groups. These adverse effects were evident in both short-term and long-term outcomes [17, 23, 33–35]. Families residing in economically disadvantaged neighborhoods may face challenges in accessing regular medical care, leading to delayed diagnosis and treatment of CHD. These delays likely contribute to the higher rates of unplanned hospitalizations as patients present with more advanced disease stages. This study could lay the groundwork for future strategies to reduce CHD-related mortality in infants and children. By informing decision makers in public health strategic planning and tackling healthcare disparities, we hope to pave the way for improved healthcare access to improve outcomes for children with CHD.

Our study observed higher mortality rates among children with congenital heart disease (CHD) from lower-income neighborhoods compared to those from higher-income areas. This finding aligns with previous research indicating an increased mortality risk in children from lower socioeconomic backgrounds. For instance, Anderson et al. analyzed data from the Pediatric Health Information System (PHIS) between 2011 and 2015, revealing that children undergoing cardiac surgery from the lowest-income neighborhoods had a 1.2 times higher mortality rate than those from higher-income neighborhoods (95% CI, 1.03–1.35) [18]. Similarly, other population-based studies have highlighted the elevated mortality risk in children from lower socioeconomic status and minority groups [36, 37].

Additionally, our findings indicate that children from lower-income neighborhoods experience longer hospital stays compared to their higher-income counterparts. This observation is consistent with Anderson et al.’s study, which reported that children from the lowest-income neighborhoods had a 7% longer length of stay than those from the highest-income neighborhoods [18]. Single-center studies by Spigel et al. [16] and Vashist et al. [38] also reported prolonged hospitalizations among children with CHD from lower socioeconomic families, including those with single ventricle lesions. These extended hospital stays may result from factors such as delayed presentation to healthcare facilities, complications due to inadequate disease management, and limited access to appropriate post-hospitalization care and resources.

Research indicates that individuals with lower educational attainment [39] experience poorer health outcomes compared to other populations [40]. This trend is linked to significant health disparities that stem from differences in educational opportunities [41, 42]. Hence, gaining a comprehensive health advantage associated with education could play a pivotal role in mitigating health disparities and enhancing the overall well-being of children and families with CHD. Promoting awareness and educating families residing in economically disadvantaged neighborhoods on early signs and symptoms of congenital heart disease (CHD), alongside promoting regular check-ups with primary care physicians and cardiologists, could serve as a strategy to prevent unplanned hospitalizations. Empowering these families to identify potential complications early may result in more effective disease management, ultimately leading to decreased hospitalization rates and improved overall outcomes.

This study provides valuable insights into the healthcare disparities based on socio-economic conditions of children with CHD. The disparities in resource utilization among children from economically disadvantaged neighborhoods, driven by higher rates of unplanned hospitalizations and longer lengths of stay, necessitate targeted effective interventions. Healthcare disparities and worse clinical outcomes in children with some complex CHD have been recently reported by Lopez et al. [43] and Krishnan et al. [44], and our study confirms those findings on a large sample of CHD. These insights could enable policymakers and healthcare providers to collaborate in developing programs, such as educational and community outreach initiatives, aimed at improving healthcare access in economically disadvantaged areas.

In this study, we used the International Classification of Diseases, Tenth Revision (ICD-10) codes to extract data of congenital heart disease (CHD). It’s important to note that the reliance on administrative databases in this study introduces certain inherent limitations. These limitations encompass the potential for coding errors, as well as the possibility of both undercounting and overcounting cases of CHD. However, AHRQ (Agency for Healthcare Research and Quality) and HCUP (Healthcare Cost and Utilization Project) implement rigorous protocols and methodologies to ensure that the data provided is accurate prior to the release of the database. We utilized neighborhood ZIP Codes to categorize the estimated median household income of residents in a patient’s ZIP Code into four quartiles, serving as a proxy for their socio-economic status (SES). While household income is a vital aspect of SES, we were unable to incorporate other significant factors such as education and occupation. These factors are not interchangeable, however, we are limited as those factors are not included in the KID database. Additionally, we lacked individual household-level data and confined our analysis to the neighborhood level by ZIP code. Despite the limitations of using Zip Code data for SES, household income has been more commonly employed as an SES indicator in studies conducted in the United States compared to studies conducted elsewhere.

In conclusion, this study highlights the critical impact of neighborhood income on unplanned hospital admissions, outcomes, and utilization of resources among children with CHD using a nationwide database. Addressing these disparities is paramount in ensuring equitable healthcare access and improving overall outcomes for children with CHD, regardless of their socio-economic backgrounds.

## Data Availability

No datasets were generated or analysed during the current study.
